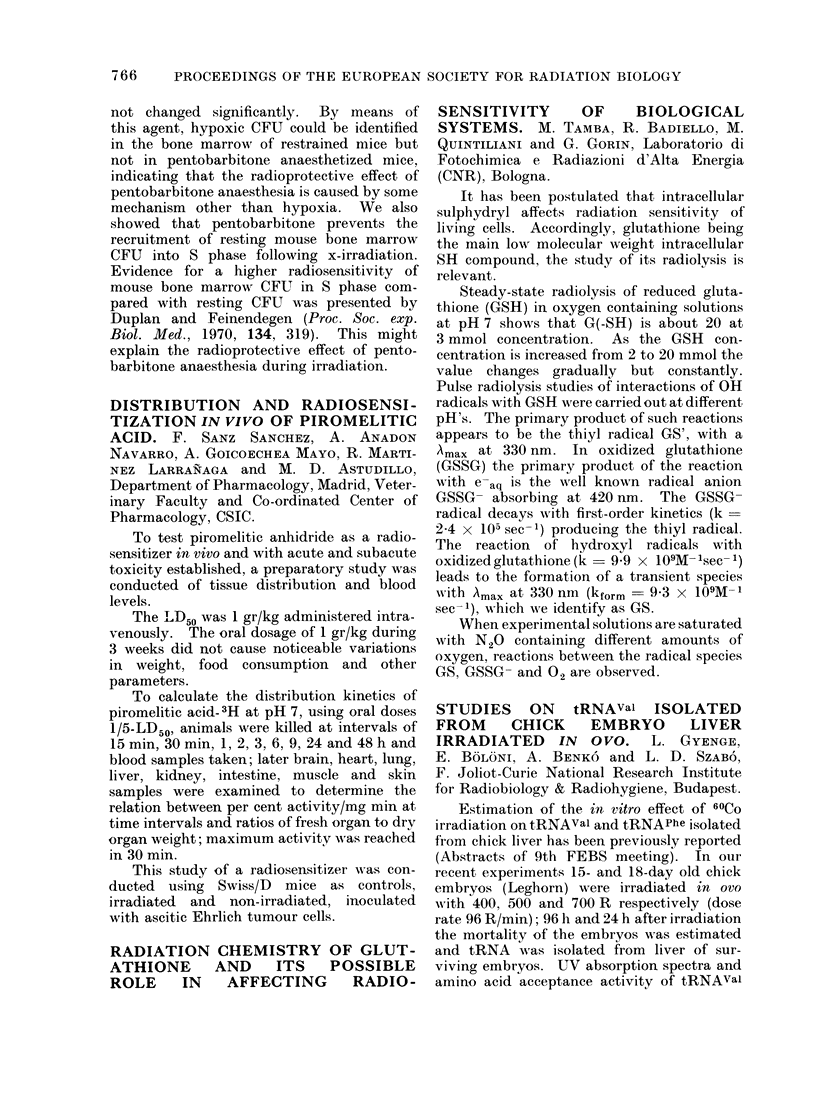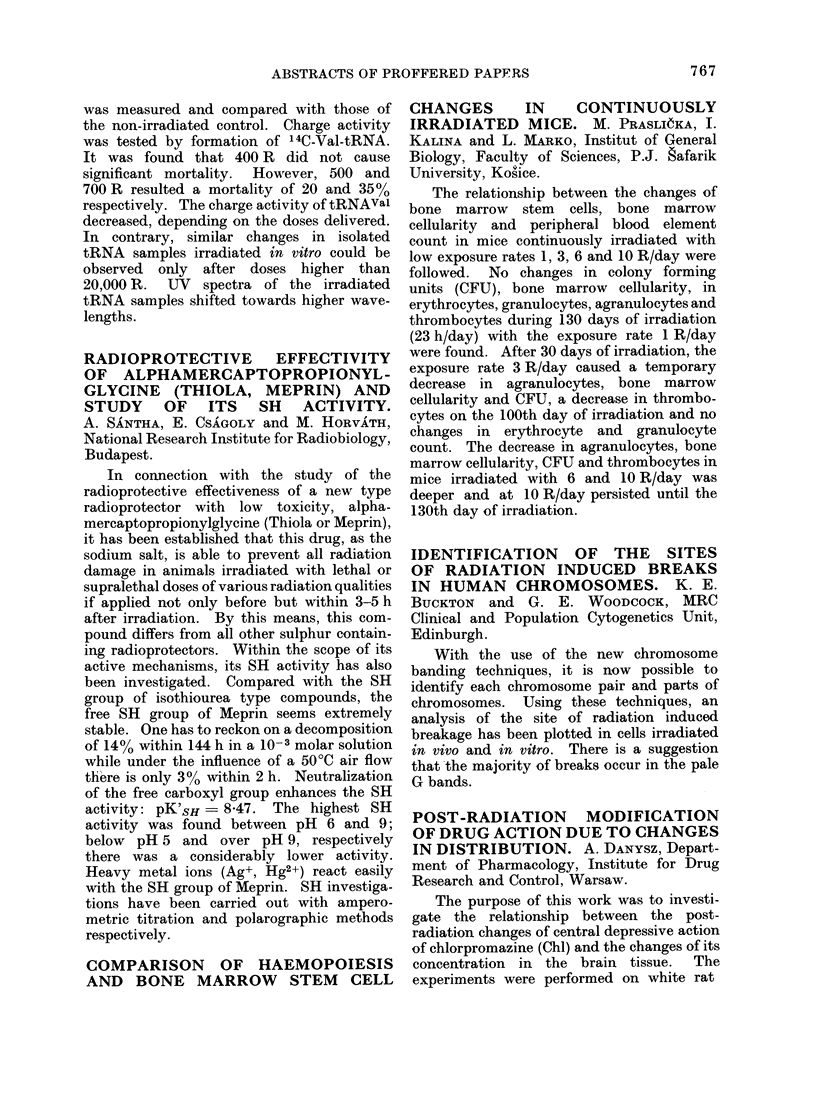# Proceedings: Studies of tRNAVal isolated from chich embryo liver irradiated in ovo.

**DOI:** 10.1038/bjc.1975.340

**Published:** 1975-12

**Authors:** L. Gyenge, E. Bölöni, A. Benkó, L. D. Szabó, F. Joliot-Curie


**STUDIES ON $tRNA^{Val}$ ISOLATED FROM CHICK EMBRYO LIVER IRRADIATED *IN OVO*.** L. Gyenge, E. Bölöni, A. Benkó and L. D. Szabó, F. Joliot-Curie National Research Institute for Radiobiology & Radiohygiene, Budapest.

Estimation of the *in vitro* effect of $^{60}Co$ irradiation on $tRNA^{Val}$ and $tRNA^{Phe}$ isolated from chick liver has been previously reported (Abstracts of 9th FEBS meeting). In our recent experiments 15- and 18-day old chick embryos (Leghorn) were irradiated *in ovo* with 400, 500 and 700 R respectively (dose rate 96 R/min); 96 h and 24 h after irradiation the mortality of the embryos was estimated and tRNA was isolated from liver of surviving embryos. UV absorption spectra and amino acid acceptance activity of $tRNA^{Val}$

was measured and compared with those of the non-irradiated control. Charge activity was tested by formation of $^{14}C$-Val-tRNA. It was found that 400 R did not cause significant mortality. However, 500 and 700 R resulted a mortality of 20 and 35% respectively. The charge activity of $tRNA^{Val}$ decreased, depending on the doses delivered. In contrary, similar changes in isolated tRNA samples irradiated *in vitro* could be observed only after doses higher than 20,000 R. UV spectra of the irradiated tRNA samples shifted towards higher wavelengths.